# In this issue

**Published:** 2023-01

**Early recognition of pulmonary complications of sickle cell disease**

Almusally presents an important review of acute and chronic pulmonary complications, including acute chest syndrome, pneumonia, pulmonary thromboembolism, pulmonary fat embolism, chronic sickle cell lung disease, and pulmonary hypertension, in patients with SCD. Bronchial asthma and obstructive sleep apnea in relation to SCD are discussed in this article. Early recognition of pulmonary complications leads to early therapeutic interventions and improvement of the overall treatment outcome. He concluded that the acute and chronic pulmonary complications of SCD cause profound morbidity and mortality. Preventive measures must be taken into consideration, and early recognition of such complications and identification of their etiologies are crucial for the management of the disease. The initiation of empirical therapy with a low threshold for blood transfusion could prevent catastrophic deterioration. Screening for PH and SCLD should be established in patients with persistent respiratory symptoms and history of repeated ACS.

***see page 10***

**Figure:**
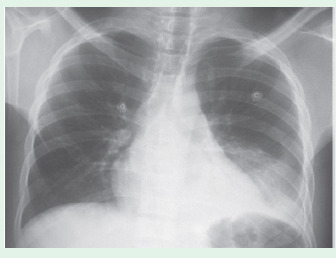
Chest X-ray demonstrate left lower lobe opacification with minimal pleural effusion in a 16 years sickle cell disease patient presented with acute chest syndrome

