# Supplementary material for: In this issue
Source: Saudi Med J. 2023 Jan;44(1):1. (PMC9987686)
Supplement: Related texts

# Abstract: Original Articles

**Primary lymphoma of the kidney.** *Pathology experience from 2 tertiary hospitals in Western Saudi Arabia*

Al-Maghrabi documents the clinicopathological features of PRL at 2 tertiary hospitals in the western region of the Kingdom of Saudi Arabia. There are 17 identified cases of PRL. The age of the patients ranges from 4-76 years (median: 50.5 years and mean: 46.8 years), 11 are males, and 6 are females. There are 12 cases of diffuse large B-cell lymphoma, 3 cases of Burkitt’s lymphoma, and 2 cases of post-transplant lymphoproliferative disorder. The median follow-up duration is 22 months. The one-year overall survival rate is 71% and the 2-year overall survival rate is 43% during follow-up. He concluded that PRL in Saudi patients are more common in males and seen in a relatively younger age group compared to the available worldwide data. The diagnosis of PRL is important to avoid tumor dissemination and unnecessary nephrectomy. Diffuse large B-cell lymphoma is the most common pathological type and non-germinal center B-cell is the most common subtype.

***see page 29***

**Figure:** Burkitt’s lymphoma expressing CD20 (immunohistochemistry stain, 200×).

**Morbidity profile among older people at primary health care centers in Saudi Arabia during the period 2012-2020**

Al-Amoud et al evaluate the morbidity profile and explore the geriatric giants, health problems, and their risk factors among old people in the older people health clinics at primary health care centers (PHCCs) in Saudi Arabia. This is a record-based descriptive cross-sectional study. Data was collected between 2012-2020 using the health data of older people to whom comprehensive geriatric assessment (CGA) was carried out at 1,481 PHCCs in Saudi Arabia. Data included sociodemographic and health related characteristics, medications, results of CGA, complete clinical examination, and laboratory results. Assessment was carried out for diabetes, hypertension, obesity, underweight, vision and hearing impairments, depression, memory and cognitive impairment, risk of falls, urine incontinence, bronchial asthma, and anemia. They concluded that The study findings highlight the importance of CGA in early detection of geriatric giants, health problems, and associated risk factors among Saudi older people.

***see page 45***
